# Detection of replicative Kashmir Bee Virus and Black Queen Cell Virus in Asian hornet *Vespa velutina* (Lepelieter 1836) in Italy

**DOI:** 10.1038/s41598-019-46565-2

**Published:** 2019-07-12

**Authors:** Maurizio Mazzei, Giovanni Cilia, Mario Forzan, Antonio Lavazza, Franco Mutinelli, Antonio Felicioli

**Affiliations:** 1 Department of Veterinary Science, Viale delle Piagge 2, University of Pisa, 56124 Pisa, Italy; 2 Virology Unit, Istituto Zooprofilattico Sperimentale della Lombardia e dell’Emilia Romagna “Bruno Ubertini”, Via Antonio Bianchi 7/9, 25124 Brescia, Italy; 3 National Reference Laboratory for Honey Bee Health, Istituto Zooprofilattico Sperimentale delle Venezie, Viale dell’Università 10, 35020 Legnaro, (PD) Italy

**Keywords:** Genomic analysis, Viral transmission

## Abstract

Information concerning the pathogenic role of honey bee viruses in invasive species are still scarce. The aim of this investigation was to assess the presence of several honey bee viruses, such as Black Queen Cell Virus (BQCV), Kashmir Bee Virus (KBV), Slow Paralysis Virus (SPV), Sac Brood Virus (SBV), Israeli Acute Paralysis Virus (IAPV), Acute Bee Paralysis Virus (ABPV), Chronic Bee Paralysis Virus (CBPV), in *Vespa velutina* specimens collected in Italy during 2017. Results of this investigation indicate that among pathogens, replicative form of KBV and BQCV were detected, assessing the spillover effect of both these viruses from managed honey bees to hornets.

## Introduction

*Vespa velutina* (Lepeletier 1836), commonly named yellow-legged hornet or Asian hornet, is a honey bee predator native of South East Asia^1^, and its activity contributes to the loss of bee colonies^2,3^.

Since the first detection in Europe of *V. velutina nigrithorax* in South West of France (Aquitaine region) in 2004^4^, the predator has spread to Spain in 2010, to Portugal in 2012, to Italy in 2013, to Germany in 2014 and more recently to Belgium, Switzerland and United Kingdom^5–11^. In Italy, the Asian hornet was firstly detected in Liguria and Piedmont regions, then it has been observed in Veneto, Lombardy and Tuscany^12,13^. The success of the rapid widespread of this invasive species in new countries is mainly due to the lack of specialist enemies^14^. Following the impact of this predator on honey bee management the European Union in 2016 has included *V. velutina* in the list of invasive alien species^15^.

The predatory activity of *V. velutina* is modulated by honey bees life cycle and carried out by catching the prey hovering in front of the beehive entrance^3,16^. In Europe, in summer, when the honey bee colony has reached the maximum population density, *V. velutina* predatory pressure increases^3,16,17^. Due to the Asian hornet predation the honey bee foraging activity is inhibited, causing an increase of colony death rate during winter^3,16^.

So far, few information are available on the presence of honey bee viruses in *V. velutina*.

Kashmir Bee Virus (KBV) is a positive sense ssRNA virus belonging to the *Dicistroviridae* family within the *Cripavirus* genus^18,19^. This virus is genetically related to Acute Bee Paralysis Virus (ABPV)^20^ and both can co-infect the same hive and the same bee^20,21^. The virus could persist at low titres in apparently healthy colonies, but the viral replication could be enhanced by the presence of honey bee stress factors triggering the loss of the colony^18,20,22,23^ resulting lethal in different developing stages of honey bee^18,23–25^. Transmission of KBV may occur by ingesting contaminated brood food such as honey, pollen, royal jelly^18,23,26–28^, or by *Varroa destructor* bite^26,29,30^. While in Europe the KBV has been rarely reported^31–35^, this virus is endemic in Australia and in the United States^36,37^. In Italy, KBV was detected for the first time in 2010, in one apiary in Tuscany, and in two sites in Lazio region (central Italy)^38^.

Black Queen Cell Virus (BQCV), belongs to the same genus and family of KBV, it is responsible of the death of honey bee queen larvae and pupae in their cells^39,40^. In queen larvae, the clinical signs consist of pale yellow appearance and the presence of a tough sac-like skin^40^. The cell wall with infected pupae become dark, giving the name at the viral agent^40^. The BQCV can be transmitted to queen brood and within nurse honey bees through glandular secretion of an infected nurse^27,41^. The virus can also infect the midgut of adult honey bees, probably transmitted by the microsporidia *Nosema apis*, increasing the BQCV spread^32,39,40^.

In *Apis mellifera*, to contrast the spread of viruses competent immune and behavioural mechanisms of defence are adopted, which could influence the fitness and colony activities. Natsopoulou *et al*. (2016) reported that DWV infection could modify honey bee polyethism schedule pace, accelerating task transitioning and increasing the fitness cost^42^. On the other hand, both KBV and BQCV seems to be not strictly related to alteration of honey bee performances^43,44^.

The KBV has been detected also in *Vespula vulgaris* and *V. germanica*^45–47^. Fitness in *V. vulgaris*, evaluated by nest size, has been directly linked to gradient of polyandry. The KBV infection in *V. vulgaris* enhances immune-related gene expression, which in colonies with low genetic diversity (low polyandry) determines smaller nest size and therefore a reduced fitness^48–50^.

In their natural habitat, *V. velutina* and its prey *Apis cerana* have been found infected by replicative Israeli Acute Paralysis Virus (IAPV)^51^. The Asian hornet infection by IAPV seems to be related to the ingestion of infected honey bees^51^. Moreover, in South West of France, the replicative forms of Sac Brood Virus (SBV), Moku virus and IAPV were detected in *V. velutina* specimens^51–53^. Recently, in Italy, both *Vespa crabro* and *V. velutina* were found infected by replicative form of Deformed Wing Virus (DWV), with the former showing clinical evidence (deformed wing) of infection^54–56^. The aim of this investigation was to assess the presence of BQCV, KBV, ABPV, SBV, IAPV, Slow Paralysis Virus (SPV) major, SPV minor, Apis Iridescent Virus (AIV), Chronic Bee Paralysis Virus (CBPV) in *V. velutina* specimens collected in Italy during 2017.

## Material and Methods

### Sampling

*V. velutina* specimens were sampled in Liguria region (North West Italy) from late April to mid-November 2017. From 30^th^ April to 30^th^ May (named early-season) fifteen workers were sampled in Airole area (43°52′26.8″N 7°33′00.3″E). On the 24^th^ July (named mid-season) fifteen workers were collected in Bordighera (43°46′45.2″N 7°39′50.3″E), Sanremo (43°49′24.8″N 7°44′24.2″E) and Dolceacqua (43°51′25.5″N 7°37′20.3″E) areas. On 15^th^ November (named late-season), six newly-emerged specimens (three fermales and three males) were sampled in Ventimiglia within the botanic garden “Giardini Hanbury” (43°46′57.9″N 7°33′14.7″E). For the early-season and mid-season sampling, the hornets have been collected in front of the apiaries during their predatory activity. While, the late-season samples were collected directely from their nest. The caste of the three newly-emerged females collected in the late season was determined by performing the wet weight measure^57^.

### Total RNA extraction

Total RNA was extracted from 6 pools (each composed of 5 individuals), three pools belonging to early-season for a total of 15 specimens (Airole) and three (5 specimens each) from mid-season sampling (Sanremo, Bordighera and Dolceacqua respectively). RNA from 6 late-season specimens (Ventimiglia) was extracted from each individual. This different extraction approach was used in order to differentiate the viral presence between the only three male and three female specimens and to evaluate the potential infection in the reproductive caste (gynes and drones).

Total RNA extraction procedure was performed as previously described^56^. Briefly, total RNA was obtained by RNeasy Mini Kit (Qiagen, Hilden, Germany) following tissue homogenization using a TissueLyser II (Qiagen) carried for 3 minutes at 25 Hz. Samples were eluted in 30 µl RNase-free water, quantified by Qubit using the RNA HS assay kit (Life-Technologies, Stafford, USA) and stored in aliquots at −80 °C until use. As a negative control, RNA obtained from a fly (*Musca domestica*) was used.

### PCR assays

All extracted RNAs were retro-transcribed by M-MLV Reverse Transcriptase (Invitrogen, Carlsbad, USA), using a blend of oligo-d (T) primers and random hexamers following manufacturer’s instruction. Five microliters of the obtained cDNAs were used as a template for the PCR reactions, which were carried out with HotStarTaqPlus Polymerase Mix (Qiagen).

Primers to amplify viral genomes of the honey bee viruses here investigated are reported in Table 1.

**Table 1:** List of primers used to detect viruses in *Vespa velutina* specimens.

Target	Primer Name	Sequence (5′-3′)	Reference
KBV	KBV 83F	ACCAGGAAGTATTCCCATGGTAAG	^79^
	KBV 161R	TGGAGCTATGGTTCCGTTCAG	
ABPV	APV 95F	TCCTATATCGACGACGAAAGACAA	^79^
	APV 159R	GCGCTTTAATTCCATCCAATTGA	
IAPV	IAPV B4S0427_R130M	RCRTCAGTCGTCTTCCAGGT	^80^
	IAPV B4S0427_L17M	CGAACTTGGTGACTTGARGG	
BQCV	BQCV 9195F	GGTGCGGGAGATGATATGGA	^79^
	BQCV 8265R	GCC GTC TGA GAT GCA TGA ATA C	
SBV	SBV 311F	AAGTTGGAGGCGCGyAATTG	^79^
	SBV 380R	CAAATGTCTTCTTACdAGAGGyAAGGATTG	
CBPV	CPV 304F	TCTGGCTCTGTCTTCGCAAA	^79^
	CPV 371R	GATACCGTCGTCACCCTCATG	
SPV major	SPV 8383F	TGATTGGACTCGGCTTGCTA	^81^
	SPV 8456R	CAAAATTTGCATAATCCCCAGTT	
SPV minor	SPV Minor F1	ATAGCGCTTTAGTTCAATTGCCAT	^82^
	SPV Minor R1	CTGGAATATGACCATCACGCAT	

Samples giving positive results to PCR were sequenced (BMR genomics, Padova) and results analysed by BLAST^58^. Phylogenetic analysis was performed by the Maximum Likelihood method based on the Tamura-Nei model using MEGA software^59^.

### Strand-specific RT-PCR

The replication activities of detected viruses were evaluated by strand specific RT-PCRs using specific primers as previously described^60^. All cDNAs were amplified by PCR for the related viral target. Amplicons were visualised on a 2% agarose gel and confirmed by sequence analysis (BMR Genomics, Padova).

### Negative staining electron microscopy (nsEM)

The three females sampled in the late season were used for nsEM analyses. The extracts were prepared and treated using the method commonly applied for honey bees^61^. Each individual was placed in 2.4 ml 0.001 M potassium buffer (PB), pH 6.7, containing 0,2% sodium diethyldithiocarbamate (DIECA) to prevent melanization and then mechanically homogenized (Ultraturrax – Ika Werk, Staufen, Germany). The extracts were emulsified with 0.3 ml chloroform and 0.3 ml diethyl ether, and then cleared by low-speed centrifugation at 4500 g for 30 min. The supernatants were separated and again centrifuged at 9500 g for 30 min. Next, 100 μl of each supernatant was ultracentrifuged with an Airfuge (Beckman, Indianapolis, USA), operating at 21 psi 82000 g for 15 min, and fitted with an A100 rotor holding six 175 μl test tubes equipped with specific plastic adapters which permit to directly pelleting virions onto 3 mm carbon-coated Formvar copper grids. The grids were negatively stained with 2% sodium phosphotungstate (NaPT) at pH 6.8 for 90 seconds and observed with a FEI Tecnai G2 Biotwin transmission electron microscope (FEI Company, Hillsboro, USA) operating at 85 kV at 16500–43000 magnifications.

## Results

### Caste determination of late-season newly-emerged females

Among the six late-season specimens (three females and three males) sampled in Ventimiglia, the wet weight of females resulted in 420 mg, 487 mg and 517 mg respectively, heavier than the 386.4 mg recorded as highest wet weight in *V. velutina* workers collected in July. Such results indicate these individuals as gynes^57^.

### Detection of viral pathogens in *V. velutina*

No amplicons were detected for SBV, IAPV, ABPV, CBPV, SPV major and SPV minor in all *V. velutina* specimens.

**Figure 1:**
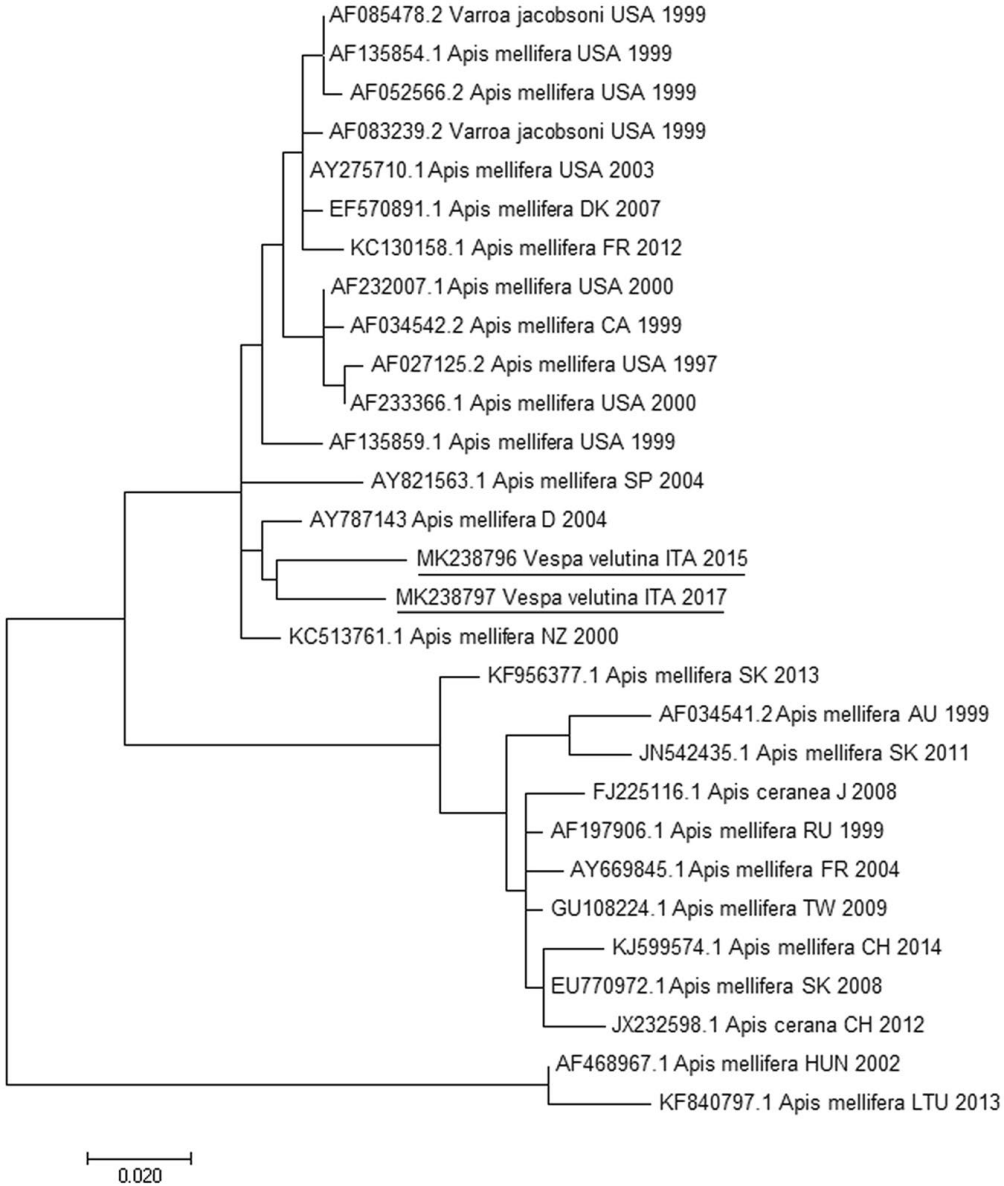
Molecular Phylogenetic analysis for RNA-dependent RNA polymerase of Kashmir Bee Virus (KBV) by Maximum Likelihood method. The evolutionary history was inferred by using the Maximum Likelihood method based on the Tamura-Nei model. The branch lengths of the tree measured the number of substitutions per site. The analysis involved 28 nucleotide sequences. There were 255 positions in the final dataset. Accession number, host, state and year of available GenBank KBV sequences are shown. The KBV sequences obtained from Italian specimens are underlined. (USA: United States of America, DK: Denmark, F: France, CA: Canada, SP: Spain, D: Germany, ITA: Italy, SK: Slovakia, AU: Austria, J: Japan, RU: Russia, TW: Taiwan, CH: China, HUN: Hungary, LTU: Lithuania).

**Figure 2:**
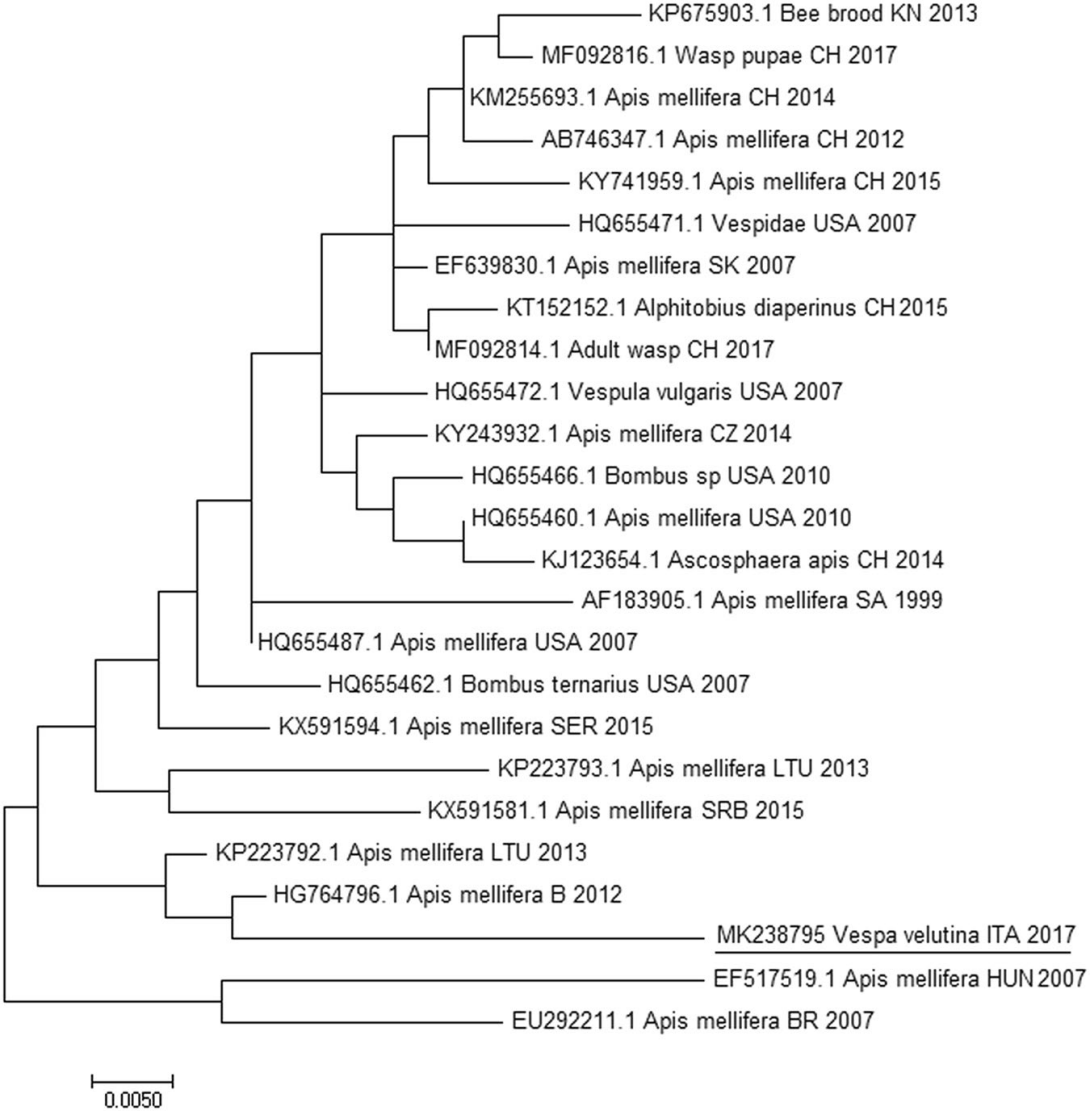
Molecular Phylogenetic analysis for capsid protein of Black Queen Cell Virus (BQCV) by Maximum Likelihood method. The evolutionary history was inferred by using the Maximum Likelihood method based on the Tamura-Nei model. The branch lengths of the tree measured the number of substitutions per site. The analysis involved 28 nucleotide sequences. There were 255 positions in the final dataset. Accession number, host, state and year of available GenBank KBV sequences are shown. The BQCV sequence obtained from Italian specimen is underlined. (KN: San Kitts and Nevis, Ch: China, USA: United States of America, SK: Slovakia, CZ: Czech Republic, SA: Saudi Arabia, SRB: Serbia, LTU: Lithuania, B: Belgium, ITA: Italy, DK: Denmark, F: France, CA: Canada, SP: Spain, D: Germany, ITA: Italy, HUN: Hungary, BR: Brazil).

Concerning BQCV, samples collected in early-season (Airole) resulted negative, while the three pools of mid-season and only the three gynes of late-season scored positive. Concerning KBV, early- and mid-season samples resulted negative, while, in late-season, one out of three males and all the gynes scored positive. It is noteworthy that all gynes resulted positive to both KBV and BQCV. The strand-specific PCR demonstrated active viral replication of KBV and BQCV (Table 2) in late season samples. Blast analysis on sequences obtained on KBV and BQCV positive amplicons performed on all PCR positive samples confirmed the specificity of the results (Tables 3 and 4). Phylogenetic analysis for both KBV and BQCV identifies a close relationship to recent European *A. mellifera* virus sequences (Figs 1 and 2).

**Table 2:** KBV and BQCV copies detected by RT-qPCR and positive/negative strand specific RT-PCR in pools of *Vespa velutina* samples collected in early- mid- and late-seasons.

Sampling Season	Sample	KBV	Strand Specific KBV RT-PCR	BQCV	Strand Specific BQCV RT-PCR
Early-season	Pool 1	NEGATIVE	−	NEGATIVE	−
	Pool 2	NEGATIVE	−	NEGATIVE	−
	Pool 3	NEGATIVE	−	NEGATIVE	−
Mid-season	Pool Dolceacqua	NEGATIVE	−	POSITIVE	ND
	Pool Bordighera	NEGATIVE	−	POSITIVE	ND
	Pool Sanremo	NEGATIVE	−	POSITIVE	ND
Late-season	Female 1	POSITIVE	ND	POSITIVE	+
	Female 2	POSITIVE	−	POSITIVE	+
	Female 3	POSITIVE	+	POSITIVE	+
	Male 1	POSITIVE	+	NEGATIVE	−
	Male 2	NEGATIVE	−	NEGATIVE	−
	Male 3	NEGATIVE	−	NEGATIVE	−

Note: (ND) not determined, (−), negative sample; (+), detection of positive and negative strand of DWV, indicative of viral replication.

**Table 3:** BLAST results on Kashmir Bee Virus (KBV) for RNA-dependent RNA polymerase consensus sequence.

Accession number	E value	Identity %	Query cover	Host	Country	Year
KC513761	3e-117	97.64	99	*Apis mellifera*	NZ	2000
AY787143	4e-116	97.24	99	*Apis mellifera*	D	2004
EF570891	2e-114	96.85	99	*Apis mellifera*	DK	2007
AF135859	2e-114	96.85	99	*Apis mellifera*	USA	1999
AF093457	2e-114	96.85	99	*Varroa jacobsoni*	USA	1999
AY275710	8e-113	96.46	99	*Apis mellifera*	USA	2003
AF200331.1	8e-113	96.46	99	*Varroa jacobsoni*	USA	1999
AF034542.2	8e-113	96.46	99	*Apis mellifera*	USA	1999
KC130158	8e-113	96.09	100	*Apis mellifera*	F	2012
AF233366	8e-113	96.09	100	*Apis mellifera*	USA	2000

Note: Accession number of reference sequences scoring the best matches by analysis for *V. velutina* KBV RNA-dependent RNA polymerase gene *consensus* sequence (MK238797). Blast scores are reported related to host, state and year of identification. For set of sequences referable to same geographical origin and host, only the one with the best score has been considered. (NZ: New Zealand, D: Germany, DK: Denmark, USA: United States of America, F: France).

**Table 4:** BLAST results on Black Queen Cell Virus (BQCV) for capsid protein *consensus* sequence.

Accession number	E value	Identity %	Query cover	Host	Country	Year
MH899990	0	97.17	100	*Apis mellifera carnica*	SL	2018
HG764796	0	96.96	100	*Apis mellifera*	B	2012
HG764797	0	96.74	100	*Heriades truncorum*	B	2012
MH900012	0	96.52	100	*Bombus pascuorum*	SL	2017
KP223792	0	96.52	100	*Apis mellifera*	LTU	2013
MH900016	0	96.30	100	*Bombus terrestris*	SL	2017
MH900011	0	96.30	100	*Bombus lapidarius*	SL	2017
KX591581	0	96.30	99	*Apis mellifera*	SRB	2015
HQ655487	0	95.22	100	*Apis mellifera*	USA	2007
HQ655467	0	95	100	*Polistes metricus*	USA	2007

Note: Accession number of reference sequences scoring the best matches by analysis for *V. velutina* BQCV capsid protein *consensus* sequence (MK238795). Blast scores are reported related to host, state and year of identification. For set of sequences referable to same geographical origin and host, only the one with the best score has been considered. (B: Belgium, LTU: Lithuania, SL: Slovenia, SRB: Serbia, USA: United States of America).

### Negative staining electron microscopy (nsEM)

**Figure 3:**
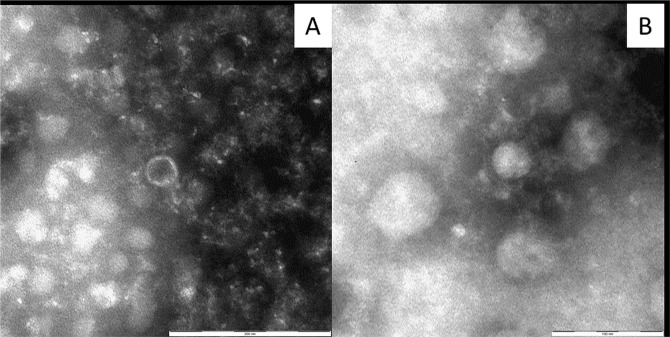
Microphotograph of Dicistrovirus-like particles observed by negative staining electron microscopy. One empty (**A**) and one full (**B**) roundish particle around 35 nm in diameter are visible. Staining NaPT 2%.

The nsEM analysis highlighted the presence of few scattered particles, morphologically closely resembling virions in all the three specimens examined. Observed particles were roundish, around 35 nm in diameter (Fig. 3) and mainly empty revealing a sharp rim; thus, their shape and size were compatible with those described for Dicistroviruses. Since these morphological characters are the same for KBV and BQCV but no specific antisera were available to perform immunoelectron microscopy (IEM), it was not possible to confirm the nature of the particles observed as virus and to exactly identify them.

## Discussion

In view of a global control strategy for the recent spread of the invasive *V. velutina* in European countries^15,62,63^, studies on the relationship among pathogens and the predator assume great significance. In this study, the presence of viruses previously detected (1993 and 2010) by Lavazza and colleagues^64^ and Porrini and colleagues in honey bees^31^ has been assessed in *V. velutina* specimens in 2017. Results indicate that pools of samples collected in mid-season resulted positive for BQCV only, while late-season samples scored positive for both KBV and BQCV. The lack of positive results for KBV and BQCV in early-season *V. velutina* imagoes and the positivity in late-season samples, suggest the absence of vertical transmission, as well as the possibility that both viruses were not circulating in the honey bees of investigated apiares in the previous season (2016). The presence of KBV and BQCV in the same specimens, indicates a possible viral co-infection in *V. velutina*, as previously described in honey bees and other hosts^20,21,65^. The detection of replications competent KBV and BQCV in late-season samples indicate that both viruses are adapted to *V. velutina* and thus can possibly infect other species, as already described for DWV^54,56^.

Data obtained by NsEM analysis, indicate that the particles observed could be effectively referable to dicistrovirus (KBV and BQCV), as indicated by sequence analysis. However, due to their overlapping morphology, it was not possible to exactly define which dicistrovirus was present. In fact, only by using IEM with specific antisera it might be possible to immune-aggregate particles, thus obtaining both an enrichment of the sample and a true viral species identification within the same genus. Nevertheless, since molecular methods can only indicate the presence of genomic material but do not prove the existence of mature “complete” virions, the detection by nsEM of particles with morphological pattern typical of either viruses could be considered a further indication of the presence of replicative forms. This hypothesis is enforced by the evidence that such nsEM analysis was performed on newly-emerged specimens that cannot have had yet the possibility to be contaminated with bee viruses during predation, but only by being fed during their larval stage with infected honey bees foragers captured by hornet workers.

Both KBV and BQCV have been identified primarily in *A. mellifera* but also in other Apoidea. The BQCV genome has been detected in *Bombus huntii*^66,67^, *B. atratus*^68,69^, *Osmia cornuta*, *O. bicornis*, *Andrena vaga, Heriades truncorum*^70^, *B. terrestris*^71–76^, *B. pascuorum*^77^, *Xylocopa virginica*^78^*, B. ignitus*^72^, *B. impatiens*^47,78,79^*, B. lucorum*^73,80^*, B. vagans*^78^ and *B. ternarius*^47,78,79^. However, BQCV replicative forms were described only in *B. huntii*^66,67^. Concerning KBV, it has been detected in *B. ignitus*^72^*, B. impatiens*^47,78,79^ but the replicative form only in *B. terrestris*^71–76^. Excluding Apoidea superfamily, to the best of Authors’ knowledge, KBV was found in *V. germanica* and *V. vulgaris*^45–47^ while BQCV has been detected only in *Vespula* spp.^78^.

Sequence analysis of KBV and BQCV from *V. velutina* indicates high identity rates to viral sequences identified in *A. mellifera*. Considering the predatory activity of *V. velutina versus A. mellifera*, this genetic similarity suggests a possible horizontal transmission route of these pathogens by ingestion of infected honey bees^18,23,26–28^. Moreover, the molecular phylogenetic analysis of KBV and BQCV from *V. velutina* identifies a close relationship to recent European *A. mellifera* virus sequences, therefore excluding the involvement of viruses of Asiatic origin.

Moreover, the relatively low number of particles observed, that, according to the established detection limits of the nsEM Airfuge method here applied, should be around 10^3^–10^4^ particles/ml, is suggestive of subclinical infection, i.e. the situation normally detected also in normoreactive/healthy honey bees^81^. In fact, by using the same nsEM Airfuge method in comparison with quantitative RT-qPCR and a sandwich Mab-based ELISA for the detection of DWV it was previously shown that the viral load in clinically affected *A. mellifera* is usually considerably higher i.e. >10^6^ viral copies^82^.

In Italy, only three KBV infected apiaries have been previously detected: one in Tuscany region nearby Liguria, and two in Lazio region^38^. At the light of the detection of KBV within this study, a retrospective analysis performed on 2015 archived *V. velutina* workers maintained at −80 °C, collected in Liguria region in Taggia area (43°51′53.1″N; 7°50′57.2″E), indicated a previous circulation of KBV (GenBank - MK238796). A possible explanation for KBV presence in this area could be the high rate of migratory beekeeping activity from other Italian sites to Liguria. It is likely that these “introduced” migratory colonies were asymptomatically infected by KBV, that could be transmitted between colonies during migration and foraging activity related to honey flow^83^, thus increasing KBV presence and making it accessible to predators such as *V. velutina*.

The positivity of only newly-emerged females collected in November could be related to the higher incidence of KBV in honey bees in the late autumn season^84^. Similarly, the presence of BQCV in Asian hornets both in mid-season and in late-season samples could be related to the high frequency of this virus in honey bee during summer^84^.

The BQCV was detected exclusively in gynes, while KBV in both male and female individuals. The small dimension of the late-season samples does not allow formulating a conclusive hypothesis on the sex-distribution of KBV and BQCV infection. Finally, variation of incidence of KBV and BQCV detected in *V. velutina* throughout the season is compatible with the increasing trend of infection usually found in the honey bee colonies^84–88^. Therefore, in late summer/early autumn there is a higher infection rate of *V. velutina* in larvae following their feeding with infected honey bee thoraxes.

In conclusion, the results of this investigation indicate that the honey bee pathogens KBV and BQCV could successfully infect *V. velutina*, although in an asymptomatic form. Additional studies should be performed *in vitro* to clarify if KBV and BQCV infection could have clinical evidence in *V. velutina*, as well as the possibility that these viruses could be transmitted vertically, in order to discuss the hypothetical role of honey bee viruses in invasive species.
